# Supplementary material for: Novel Y RNA-Derived Fragments Can Differentiate Canine Hepatocellular Carcinoma from Hepatocellular Adenoma
Source: Animals (Basel). 2023 Sep 28;13(19):3054. doi: 10.3390/ani13193054 (PMC10571523; doi:10.3390/ani13193054)
Supplement: Supplementary file 1 [file animals-13-03054-s001.zip › animals-2570765-supplementary.pdf]

Novel Y RNA-Derived Fragments Can Differentiate Canine Hepatocellular Carcinoma from Hepatocellular Adenoma

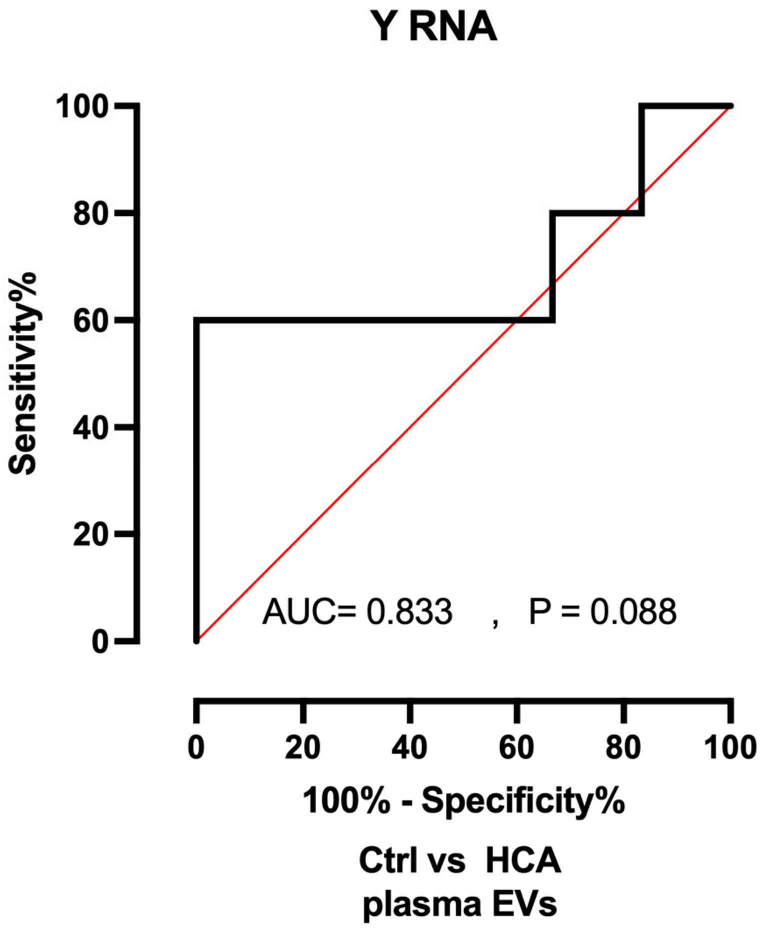

Figure S1. ROC curves of Y RNA. ROC curve (plasma Evs)) of the Y RNA for differentiating HCA (n=5) compared to the ctrl (n=6). Ctrl; control, HCA; Hepatocellular adenoma, EVs; Extracellular vesicles.
